# Effects of Nigella sativa Oil Rinsing on Salivary pH, Volume, and Protein Concentration: A Pilot Repeated-Measures Study

**DOI:** 10.7759/cureus.112590

**Published:** 2026-07-13

**Authors:** Indra Mustapha, Marzia Mustamand, Rochelle Watson, Xinbin Gu

**Affiliations:** 1 Department of Oral Pathology, Howard University, Washington, DC, USA

**Keywords:** medicine, nigella sativa, oral pathology, pathology, salivary physiology

## Abstract

Background

This study evaluated the effects of saline and *Nigella sativa* oil rinsing on salivary pH, volume, and protein concentration at different rinse durations and assessed their potential implications for oral homeostasis. Rinse durations will be extended to reflect the timeframes commonly used by patients during oil-pulling practices.

Methodology

A total of 10 healthy adults (two males, eight females) participated in this repeated-measures pilot study. Each participant served as their own control, completing saline and *N. sativa* oil rinsing protocols at one-, five-, and ten-minute durations. Saliva samples were collected after each rinse for pH and volume analysis. Salivary protein concentration was determined using NanoDrop spectrophotometry and reported descriptively. Salivary pH and volume data were analyzed using two-way repeated-measures analysis of variance (ANOVA) with Šídák multiple-comparisons testing. Statistical significance was established at a p-value <0.05.

Results

Salivary pH demonstrated a significant effect of rinse duration (F(1.775,31.96) = 10.09, p = 0.0006), but no significant treatment effect (p = 0.5000) or time-by-treatment interaction (p = 0.5641). Salivary volume demonstrated significant effects of rinse duration (F(1.804,32.48) = 176.2, p < 0.0001), treatment (F(1,18) = 8.738, p = 0.0085), and time-by-treatment interaction (F(1.804,32.48) = 50.10, p < 0.0001). Post hoc analysis identified greater salivary volume following *N. sativa* rinsing compared with saline at the one-minute rinse duration (adjusted p < 0.0001), with no significant differences observed at five or ten minutes. Mean salivary protein concentrations were descriptively higher following *N. sativa* rinsing across all rinse durations, demonstrating fold increases of 5.04×, 2.83×, and 2.51× at one, five, and ten minutes, respectively.

Conclusions

Rinse duration significantly influenced salivary pH and volume, while *N. sativa *oil produced an early increase in salivary secretion compared with saline. These findings provide preliminary evidence that *N. sativa* oil may influence short-term salivary physiology and support further investigation into its potential role in oral homeostasis.

## Introduction

Saliva is an essential biological fluid that contributes significantly to both oral and systemic health through its lubricating, buffering, antimicrobial, and immunologic functions [1-4]. Given its central role in maintaining oral homeostasis, there is growing interest in understanding how oral care products, particularly plant-derived oil rinses, influence salivary physiology [5]. A recent randomized controlled trial among 253 adults demonstrated that essential oil-containing mouthrinses were well tolerated and preserved normal salivary flow and pH, with no evidence of dry mouth following use [5].

Beyond conventional mouthrinses, natural plant-derived oils have also been investigated for their potential effects on salivary function and oral health outcomes [6]. Another randomized controlled study evaluating sesame and virgin coconut oil-pulling practices in children with gingivitis found that these interventions improved salivary buffering, with effects comparable to chlorhexidine rinsing [6]. These findings suggest that plant-derived oils may affect salivary properties and contribute to oral homeostasis [6]. Likewise, a pilot study with standardized sunflower oil increased salivary production while also reducing the oral microbial burden of the oral cavity compared to saline pulling [7]. Collectively, the current literature does indicate that plant-derived oil rinses may influence salivary physiology while promoting oral homeostasis [5-7].

*Nigella sativa *is among these plant-derived oils and has been extensively used in traditional medicine as a natural therapeutic agent [8,9]. Our previous study demonstrated that *N. sativa* possessed growth-inhibitory effects against *Streptococcus mutans* in vitro, supporting its potential as a natural antimicrobial agent in oral health applications [10]. Furthermore, preclinical evidence suggests that *N. sativa* oil preserved salivary secretion and enhanced salivary gland stem cell recovery after a radiation-induced injury [11]. This suggests that *N. sativa *oil possesses benefits that extend beyond growth-inhibitory functions and antimicrobial and anti-inflammatory activity [11-13].

Despite growing interest in the therapeutic applications of *N. sativa*, evidence of its direct effects on salivary physiology remains limited, particularly regarding the immediate effects of *N. sativa* oil rinsing on salivary pH, salivary volume, and salivary protein concentration [8,14]. This represents an important knowledge gap because modulation of these parameters may influence oral buffering capacity, microbial colonization, and oral tissue homeostasis [2,8,14]. Furthermore, understanding the time-dependent physiologic response to *N. sativa* rinsing may provide insight into its potential utility as a supportive adjunct in preventive oral health strategies [2,8,14]. Evaluating these effects across varying rinse durations may therefore help elucidate whether *N. sativa *oil induces transient or sustained physiologic changes within the oral environment, representing a novel contribution to the current literature [8,14].

Accordingly, this pilot study evaluated the effects of saline and *N. sativa* oil rinsing on salivary pH, volume, and descriptive protein concentration outcomes at one-, five-, and ten-minute durations using a within-subject repeated-measures design. We hypothesize that rinsing with *N. sativa* oil would either modulate salivary pH, volume, and protein concentration or, at a minimum, preserve these parameters without adverse effects, compared to saline across all rinse durations.

## Materials and methods

Study participants

This pilot study included 10 participants (two males and eight females) aged 18-80 years. Participants were recruited and conveniently sampled from a private practice located on Capitol Hill, Washington, DC, and salivary analyses were performed at the Howard University College of Dentistry Research Department. Age range and sex distribution reflected the availability of eligible individuals during the recruitment period, with no targeted recruitment based on these characteristics.

Eligibility criteria included systemically healthy individuals without active periodontal disease, edentulous areas, or current COVID-19 infection. Individuals who smoked or were taking medications were excluded, except for oral contraceptive use. All participants were informed of the study protocol and provided written informed consent before participation.

Given the exploratory design and small sample size of this pilot investigation, prospective clinical trial registration was not performed. Participant confidentiality was maintained through the use of unique study identifiers, and written informed consent was obtained from all participants before enrollment. The results should be interpreted as preliminary, as the use of a convenience sample and limited cohort size may restrict the generalizability of the findings to broader populations.

No *a priori *sample size or power calculation was conducted, as this investigation was designed as an exploratory pilot study. The primary aim was to generate preliminary data to evaluate the feasibility of the research design and to inform future adequately powered clinical investigations.

This study received approval from the Howard University Institutional Review Board (IRB) and was conducted from July 15, 2025, to August 22, 2025. All participants provided written informed consent before study enrollment and data collection.

Study design

This study employed a conveniently sampled, fixed, repeated-measures, within-subject design in which each participant served as their own control. Participants were exposed to two rinsing conditions in a fixed order: sterile saline, followed by *N. sativa* oil. Salivary outcomes were assessed in relation to pH, volume, and protein concentration across multiple rinse durations (one, five, and ten minutes). Extended rinse periods will be used to mirror the durations typically practiced during patient oil pulling. To minimize circadian variability, all sample collections were conducted between 10:00 AM and 12:00 PM. Consecutive study visits were separated by a one-week washout period to minimize potential carryover effects.

Participants and outcome assessors were not blinded to the intervention due to the distinct sensory characteristics of the saline and *N. sativa* oil rinses. Additionally, identification of treatment conditions was necessary for proper sample processing and analysis. However, outcome measurements were performed using standardized protocols to minimize potential measurement bias.

*Nigella sativa* oil and saline administration protocol

Participants were exposed to two rinsing solutions: a commercially available *N. sativa* oil product (SprinJene Natural Organic Black Seed Oil, SprinJene, USA) and a 0.9% saline solution, which served as the control. The* N. sativa *oil was 100% pure, cold-pressed, and undiluted, with no added solvents or synthetic additives. For each rinsing condition, a standardized volume of 5 mL was used for the assigned rinse duration (one, five, or ten minutes). Participants were instructed to gently swish the solution throughout the oral cavity and between the teeth while avoiding ingestion. At the completion of the assigned rinse period, the rinse solution and saliva were expectorated into a pre-weighed 30 mL polypropylene collection tube for subsequent analysis.

Sample collection protocol, sample processing, and storage

Participants were instructed to abstain from eating or drinking for at least one hour before sample collection. No baseline unstimulated saliva sample was collected before rinsing. At each visit, unstimulated whole saliva was collected following the assigned rinsing condition. For each collection, participants performed a timed oral rinse (saline or *N. sativa* oil), after which saliva was expectorated into a pre-weighed 30 mL polypropylene test tube. Samples were then transferred into 15 mL centrifuge tubes and immediately placed on ice.

**Figure 1:**
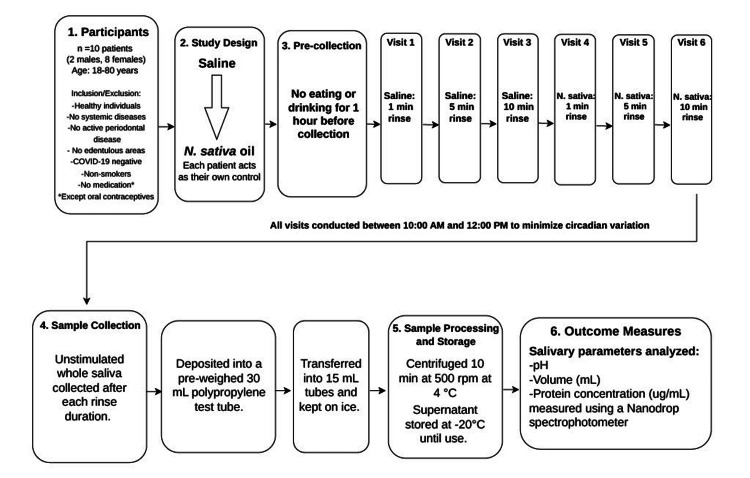
Study design, saliva collection, processing, and analysis workflow. Participants (n = 10) completed six study visits involving saline and *Nigella sativa* oil rinses at one-, five-, and ten-minute durations in a repeated-measures design in which each participant served as their own control. Unstimulated whole saliva was collected following each rinse, processed by centrifugation, stored at −20°C, and analyzed for pH, volume, and protein concentration.

Each participant completed six study visits: three visits for saline rinses (one-, five-, and ten-minute durations) and three visits for *N. sativa* oil rinses (one-, five-, and ten-minute durations), with rinse duration assigned per visit according to the study protocol. Subsequent study visits were separated by a one-week washout period to reduce carryover effects. Following collection, saliva samples were centrifuged at 500 rpm (55 × g) for 10 minutes at 4°C. The resulting supernatant was aliquoted and stored at −20°C until further analysis. Figure 1 illustrates the overall study workflow.

Outcome measures

Salivary outcomes included pH, volume, and total protein concentration. Salivary pH was measured using a calibrated digital pH meter. Before each testing session, the electrode was rinsed with distilled water, gently blotted dry, and calibrated using standard pH 7.0, 4.0, and 10.0 buffer solutions according to the manufacturer’s instructions. The electrode was rinsed with distilled water between calibration and sample measurement, and saliva pH was recorded after the reading stabilized. Total protein concentration was quantified using a NanoDrop 2000 spectrophotometer (Thermo Fisher Scientific, Waltham, MA, USA). Before analysis, the instrument was blanked with deionized water, and protein concentration was determined using the Protein A280 application and reported as μg/mL. Salivary protein concentration data were available only as mean values for each treatment and rinse duration; therefore, individual-level inferential statistical analyses were not performed. Protein concentration outcomes were reported as mean concentration (μg/mL) and fold change relative to saline controls. These findings were analyzed descriptively and should be interpreted cautiously due to the exploratory nature of this investigation. Figure 1 illustrates the overall study workflow.

Statistical analysis

Data were analyzed using GraphPad Prism (GraphPad Software Version 11.0.2, LLC, San Diego, CA, USA). Descriptive statistics were calculated for outcome measures and reported as mean ± standard deviation (SD).

Data distribution was assessed using the Shapiro-Wilk normality test before inferential analyses. Specifically, salivary volume data were log-transformed before normality assessment and subsequent repeated-measures analysis of variance (RM ANOVA). Salivary pH and salivary volume were analyzed using two-way RM ANOVA with treatment (saline versus *N. sativa* oil) and rinse duration (one, five, and ten minutes) as within-subject factors. Because each participant underwent all experimental conditions, a repeated-measures design was employed in which participants served as their own controls. Sphericity was not assumed; the Geisser-Greenhouse correction was applied to repeated-measures analyses where appropriate.

Effect sizes were calculated using partial eta-squared (partial η²) and reported for treatment, time, and interaction effects from the RM ANOVA model. Effect sizes were calculated using partial eta squared (partial η²). Effect sizes were interpreted according to commonly used thresholds, with values of approximately 0.01, 0.06, and 0.14 representing small, medium, and large effects, respectively. Statistical significance was established at a p-value <0.05.

The main effects of treatment and rinse duration, as well as the treatment × time interaction, were evaluated. When significant effects were identified, Šídák multiple-comparisons tests were performed to compare treatment groups at each rinse duration and to assess differences among rinse durations. Mean differences, 95% confidence intervals (CIs), and adjusted p-values were reported for pairwise comparisons obtained from post hoc Šídák multiple-comparisons testing following the RM ANOVA.

Salivary protein concentration data were available only as mean values for each treatment and rinse duration. Therefore, protein concentration outcomes were analyzed descriptively and reported as mean concentration (μg/mL) and fold change relative to saline controls. Inferential statistical testing was not performed for protein concentration analyses.

Data are presented as mean ± SD. Statistical significance was defined as a p-value* *<0.05. For highly significant results that exceeded the reporting precision of GraphPad Prism, p-values are reported as p <0.0001 in accordance with standard reporting practices.

## Results

Participant characteristics

A total of 10 participants (two males and eight females) between 18 and 80 years of age completed the study. All participants underwent each experimental condition and served as their own controls. No missing data were recorded.

Salivary pH

**Figure 2:**
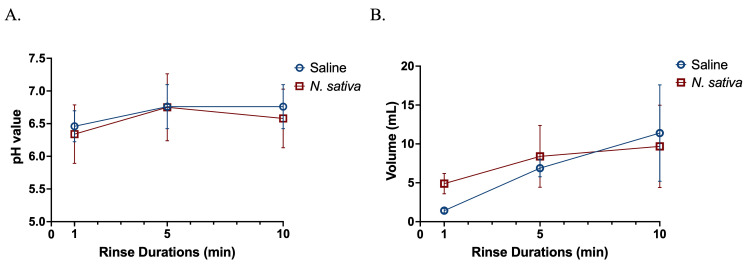
Effects of saline and Nigella sativa oil rinsing on salivary pH and volume at different rinse durations. (A) Mean salivary pH values following saline and *Nigella sativa* oil rinsing for one, five, and ten minutes. A significant overall effect of rinse duration on salivary pH was observed, whereas no significant treatment effect or treatment × time interaction was detected. (B) Mean salivary volume (mL) following saline and *Nigella sativa* oil rinsing for one, five, and ten minutes. Salivary volume increased significantly with increasing rinse duration, and a significant treatment × time interaction was observed. Data are presented as mean ± SD (n = 10).

Descriptive statistics for salivary pH following saline and *N. sativa* oil rinsing are presented in Table 1 and Figure 2, Panel A. Mean salivary pH values across all rinse durations and treatment groups are listed in Table 1. It is important to note that data distribution was assessed using the Shapiro-Wilk normality test before inferential analysis. Salivary pH data were normally distributed across most treatment and rinse duration conditions, except for the one-minute *N. sativa* oil condition (W = 0.7186, p = 0.0015). Given the exploratory pilot design, repeated-measures structure of the study, and the robustness of RM ANOVA to minor deviations from normality, RM ANOVA with the appropriate corrections was performed. Complete Shapiro-Wilk test results are provided in Appendix 1.

**Table 1:** Salivary pH and volume following saline and Nigella sativa oil rinsing. Descriptive statistics (mean ± SD) of salivary pH and salivary volume (mL) collected after one-, five-, and ten-minute rinse durations with saline and *Nigella sativa* oil (n = 10). Each participant completed all rinse conditions in a repeated-measures design.

Saliva collected	Outcome measure	Rinse duration: 1 minute		Rinse duration: 5 minutes		Rinse duration: 10 minutes	
Saline solution saliva		Mean	SD	Mean	SD	Mean	SD
	pH (1–14)	6.46	0.237	6.76	0.337	6.76	0.337
	Volume (mL)	1.43	0.292	6.88	1.12	11.4	6.20
*Nigella sativa* oil saliva	pH (1–14)	6.34	0.448	6.75	0.513	6.58	0.449
	Volume (mL)	4.89	1.31	8.39	3.97	9.69	5.30

Two-way RM ANOVA with the Geisser-Greenhouse correction (ε = 0.8877) demonstrated a significant effect of rinse duration on salivary pH (F(1.775, 31.96) = 10.09, p = 0.0006, partial η² = 0.359), indicating that salivary pH varied over time regardless of treatment (Table 2). In contrast, no significant treatment effect was observed between saline and *N. sativa* oil rinses (F(1,18) = 0.474, p = 0.5000, partial η² = 0.061), and no significant treatment × time interaction was detected (F(1.775, 31.96) = 0.546, p = 0.5641, partial η² = 0.029) (Table 2).

**Table 2:** Two-way repeated-measures ANOVA analysis of salivary pH following saline and Nigella sativa oil rinsing. Partial η² values are reported as measures of effect size. ***: p < 0.001. ANOVA = analysis of variance

Source of variation	F (DFn, DFd)	P-value	Partial η²
Time × treatment interaction	F(1.775, 31.96) = 0.546	0.5641	0.029
Time effect	F(1.775, 31.96) = 10.09	0.0006***	0.359
Treatment effect	F(1,18) = 0.474	0.5000	0.061

Post hoc Šídák multiple-comparisons testing revealed no significant differences in salivary pH between saline and *N. sativa* oil at the one-minute (p = 0.8480), five-minute (p > 0.9999), or ten-minute (p = 0.6930) rinse durations (Table 3). However, significant overall time-dependent increases in salivary pH were observed (Table 4). Mean pH values were significantly higher at five minutes compared with one minute (mean difference = −0.355, 95% CI: −0.587 to −0.123, adjusted p = 0.0023) and at 10 minutes compared with one minute (mean difference = −0.270, 95% CI: −0.497 to −0.043, adjusted p = 0.0172). No significant difference was observed between the five- and ten-minute time points (mean difference = 0.085, 95% CI: −0.091 to 0.261, adjusted p = 0.5299).

**Table 3:** Šídák-adjusted post hoc comparisons of salivary pH between saline and Nigella sativa oil rinses at each time point (one, five, and ten minutes). Adjusted p-values account for multiple comparisons using the Šídák correction. CI = confidence interval

Between-treatment comparisons at each time point			
Comparison	Mean difference (Saline − *Nigella sativa*)	95% CI	Adjusted p-value
1 minute	0.120	−0.315 to 0.555	0.8480
5 minute	0.010	−0.509 to 0.529	>0.9999
10 minutes	0.180	−0.291 to 0.651	0.6930

**Table 4:** Šídák-adjusted post hoc comparisons of overall salivary pH across time points following saline and Nigella sativa oil rinsing. Adjusted p-values account for multiple comparisons using the Šídák correction. **: p < 0.01, *: p < 0.05. CI = confidence interval

Overall time-effect comparisons			
Comparison	Mean Difference	95% CI	Adjusted p-value
1 min vs 5 min	−0.355	−0.587 to −0.123	0.0023**
1 min vs 10 min	−0.270	−0.497 to −0.043	0.0172*
5 min vs 10 min	0.085	−0.091 to 0.261	0.5299

Collectively, these findings indicate that salivary pH increased with longer rinse durations regardless of treatment type. However, *N. sativa* oil did not have a measurable effect on salivary pH compared with saline at any evaluated time point. While these findings suggest that *N. sativa *oil does not substantially alter short-term salivary acid-base homeostasis, the absence of observed treatment differences should be interpreted cautiously, given the small sample size and the possibility that the study was not powered to detect smaller treatment-related effects.

Salivary volume

Descriptive salivary volume data are presented in Table 1 and Figure 2B. It is important to note that salivary volume data were log-transformed before assessment of normality using the Shapiro-Wilk test because the untransformed data demonstrated deviation from normality. Following transformation, all salivary volume datasets met the assumption of normality across treatment conditions and rinse durations (*p* > 0.05). Complete Shapiro-Wilk test results are provided in Appendix 2.

Salivary volume increased progressively with rinse duration in both treatment groups. Mean salivary volume following saline rinsing increased from 1.43 ± 0.29 mL at one minute to 11.4 ± 6.20 mL at ten minutes, whereas *N. sativa* oil rinsing increased salivary volume from 4.89 ± 1.31 mL to 9.69 ± 5.30 mL over the same time period.

Two-way RM ANOVA performed on log-transformed salivary volume data with the Geisser-Greenhouse correction (ε = 0.9021) demonstrated significant effects of rinse duration (F(1.804, 32.48) = 176.2, p < 0.0001, partial η² = 0.907), treatment (F(1,18) = 8.738, p* *= 0.0085, partial η² = 0.548), and the treatment × time interaction (F(1.804, 32.48) = 50.10,* *p < 0.0001, partial η² = 0.736) (Table 5).

**Table 5:** Two-way repeated-measures ANOVA analysis of salivary volume following saline and Nigella sativa oil rinsing. Partial η² values are reported as measures of effect size. **: p < 0.01, ****: p < 0.0001. ANOVA = analysis of variance

Source of variation	F (DFn, DFd)	P-value	Partial η²
Time × treatment interaction	F(1.804, 32.48) = 50.10	<0.0001****	0.736
Time effect	F(1.804, 32.48) = 176.2	<0.0001****	0.907
Treatment effect	F(1,18) = 8.738	0.0085**	0.548

Post hoc Šídák multiple-comparisons testing demonstrated a significant difference between saline and *N. sativa* oil at the one-minute rinse duration, with greater log-transformed salivary volume following *N. sativa* oil rinsing (mean difference = −0.5296, 95% CI: −0.6481 to −0.4111, adjusted p < 0.0001) (Table 6). No significant differences were observed between treatments at five minutes (mean difference = −0.0567, 95% CI: −0.2199 to 0.1064, adjusted p = 0.7281) or ten minutes (mean difference = 0.0736, 95% CI: −0.1709 to 0.3181, adjusted p = 0.8229).

**Table 6:** Šídák-adjusted post hoc comparisons of salivary volume between saline and Nigella sativa oil rinses at each time point (one, five, and ten minutes). Adjusted p-values account for multiple comparisons using the Šídák correction. CI = confidence interval

Between-treatment comparisons at each timepoint			
Comparison	Mean Difference (Saline − *N. sativa*)	95% CI	Adjusted* p*-value
1 min	−0.5296	−0.6481 to −0.4111	<0.0001****
5 min	−0.0567	−0.2199 to 0.1064	0.7281
10 min	0.0736	−0.1709 to 0.3181	0.8229

Analysis of the overall time effect demonstrated significant differences across all rinse durations (Table 7). Salivary volume was significantly greater at five minutes compared with one minute (mean difference = −0.4503, 95% CI: −0.6069 to −0.2936, adjusted* *p < 0.0001), at ten minutes compared with one minute (mean difference = −0.5622, 95% CI: −0.7636 to −0.3607, adjusted p < 0.0001), and at ten minutes compared with five minutes (mean difference = −0.1119, 95% CI: −0.2064 to −0.0175, adjusted* *p = 0.0175).

**Table 7:** Šídák-adjusted post hoc comparisons of overall salivary volume across time points following saline and Nigella sativa oil rinsing. Adjusted p-values account for multiple comparisons using the Šídák correction. *: p < 0.05, ****: p < 0.0001. CI = confidence interval

Overall time-effect comparisons			
Comparison	Mean difference	95% CI	Adjusted p-value
1 minute vs. 5 minutes	−0.4503	−0.6069 to −0.2936	<0.0001****
1 minute vs. 10 minutes	−0.5622	−0.7636 to −0.3607	<0.0001****
5 minutes vs. 10 minutes	−0.1119	−0.2064 to −0.0175	0.0175*

Collectively, these findings demonstrate that salivary volume increased significantly with prolonged rinse duration and was influenced by rinse treatment. *N. sativa* oil produced a significantly greater salivary volume response during the one-minute rinse condition compared with saline, whereas differences between treatments were not observed at longer rinse durations. These findings suggest that *N. sativa* oil may induce an early transient enhancement of salivary secretion, while prolonged rinsing primarily drives the time-dependent increase in salivary volume.

Salivary protein concentration

**Figure 3:**
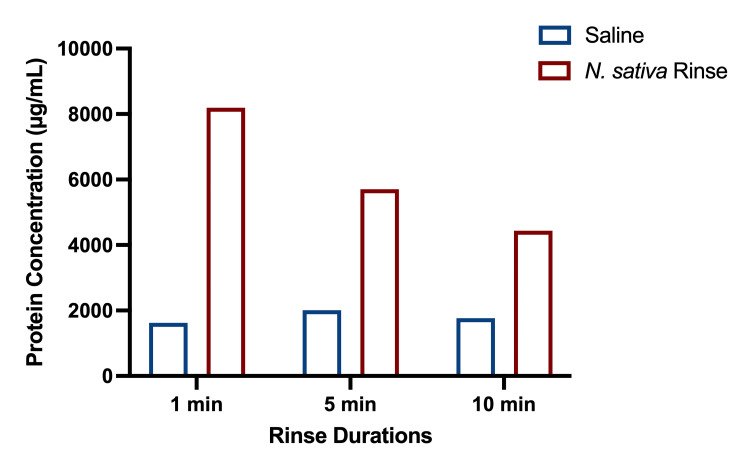
Average salivary protein concentration after saline and Nigella sativa oil rinsing. Mean salivary protein concentrations (μg/mL) measured by NanoDrop spectrophotometry following one-, five-, and ten-minute rinses with saline and *Nigella sativa* oil. *Nigella sativa* oil demonstrated higher average protein concentrations than saline at all rinse durations. Data are presented as descriptive means.

Average salivary protein concentrations following saline and *N. sativa* oil rinsing are presented in Table 8 and Figure 3. Across all rinse durations, *N. sativa* oil was associated with higher average salivary protein concentrations than saline.

**Table 8:** Descriptive salivary protein concentrations and fold change following saline and Nigella sativa rinse. Protein concentrations were consistently higher following *Nigella sativa* rinse compared with saline across all timepoints, with the largest relative increase observed at one minute.

Timepoint	Saline (μg/mL)	*Nigella sativa* rinse (μg/mL)	Fold change (*Nigella sativa*/Saline)
1 minute	1,627.1	8,193.4	5.04×
5 minutes	2,012.3	5,706.9	2.83×
10 minutes	1,765.6	4,436.1	2.51×

At the one-minute rinse duration, mean protein concentration increased from 1,627.1 μg/mL with saline to 8,193.4 μg/mL following *N. sativa* oil rinsing, representing a 5.04-fold increase. At five minutes, protein concentration increased from 2,012.3 μg/mL to 5,706.9 μg/mL (2.83-fold increase), while at ten minutes, protein concentration increased from 1,765.6 μg/mL to 4,436.1 μg/mL (2.51-fold increase).

Only aggregate mean values were available for protein concentration measurements; inferential statistical analyses were not performed. Protein concentrations were analyzed descriptively and should be interpreted cautiously due to the exploratory nature of this study.

## Discussion

The present pilot study evaluated the short-term effects of *N. sativa *oil rinsing on salivary pH, volume, and protein concentration compared with saline rinsing across varying rinse durations. The findings demonstrated that rinse duration significantly influenced salivary pH and volume, while *N. sativa *oil produced a distinct early increase in salivary volume compared with saline. Salivary protein concentrations were descriptively higher following *N. sativa *oil rinsing, although these findings require cautious interpretation. It is critical to note that this study is exploratory in nature and is not intended to provide clinical recommendations or guide individual patient care. Rather, the findings should be assessed with caution due to the lack of generalizability. These outcomes are intended to generate preliminary data to inform and support a future controlled clinical investigation.

One of the principal findings of this study was that salivary pH increased significantly over time regardless of treatment type. Both saline and *N. sativa* oil rinses produced modest elevations in pH between the one-minute and subsequent rinse durations; however, no significant differences were observed between treatments at any individual time point. These findings suggest that prolonged oral stimulation itself may contribute more substantially to short-term alterations in salivary pH than the specific rinsing agent used. This observable effect is biologically plausible as increased salivary flow is known to enhance bicarbonate secretion, thereby elevating salivary buffering capacity and pH [15,16]. The observed rise in pH across rinse durations is therefore consistent with established physiologic mechanisms of stimulated salivary secretion. Importantly, *N. sativa* oil did not adversely affect salivary pH in our exploratory investigation, suggesting that its use as an oral rinse is unlikely to modify acid-base homeostasis severely within the oral environment. This coincides well with other studies in the literature demonstrating that* N. sativa* provides beneficial effects and, at a minimum, does not appear to be detrimental to oral homeostasis [8,17]. Previous research has specifically evaluated the effects of *N. sativa*’s exposure on salivary pH [17]. A clinical study examining the effects of chewing *N. sativa* seeds in healthy participants demonstrated a significant increase in salivary pH following exposure, suggesting that *N. sativa* may influence salivary acid-base regulation under certain conditions [17]. However, this study evaluated whole seed chewing rather than *N. sativa* oil rinsing, and differences in formulation, exposure time, and constituent release may contribute to variations in salivary responses [17].

Interestingly, a recent in vitro study showed that a water-based extract of *N. sativa *seeds, designed to mimic oral exposure during chewing, exerted significant cytotoxic effects against oral cancer and pre-cancerous leukoplakia cells [18]. While noteworthy, this activity was primarily associated with higher levels of the water-soluble constituent α-hederin, whereas the principal bioactive constituent of *N. sativa*, thymoquinone, was detected only in modest amounts, indicating minimal release in an aqueous model [18]. These findings suggest that the extraction method, as well as the bioavailability of *N. sativa*’s active constituents, may play a major role in the beneficial activities associated with this plant-derived oil [18]. Together, these findings suggest that the formulation and bioavailability of *N. sativa*’s active constituents may play an important role in determining its biologic effects. Therefore, the lack of significant differences in salivary pH between saline and *N. sativa* oil rinsing observed in the present study may reflect differences in delivery method and constituent availability compared with other forms of *N. sativa* exposure.

It is also important to acknowledge that the absence of significant differences between saline and *N. sativa* oil for salivary pH measures may have been influenced by the small sample size of this exploratory pilot study. Consequently, the study was not adequately powered to detect small treatment effects, and these findings should therefore be assessed with caution.

The effects of *N. sativa *oil on salivary volume were particularly intriguing. A significant overall treatment effect and treatment-by-time interaction were observed, suggesting that *N. sativa* oil influenced salivary secretion differently from saline over the course of the rinsing periods. More specifically, *N. sativa* oil produced a significantly greater salivary volume than saline after the one-minute rinse, whereas no significant differences were observed at the five- or ten-minute rinse durations. This finding suggests that the salivary stimulatory effect of *N. sativa* oil may occur rapidly after exposure, but becomes less apparent with longer rinse durations as salivary secretion increases over time in both groups. This rapid salivary response after *N. sativa *oil rinsing may reflect gustatory, sensory, mechanical, or oral chemical stimulation induced by the oil and its bioactive constituents [14,19,20]. Experimental studies have demonstrated that sensory stimulation and oral exposure to chemically active compounds can transiently increase salivary flow and alter salivary composition [20].

The early increase in salivary volume observed following *N. sativa* oil rinsing suggests that the rinse may stimulate salivary gland activity, which could contribute to improved oral lubrication and clearance. Although studies directly examining the immediate effects of *N. sativa* oil on salivary secretion are limited, previous experimental work suggests that *N. sativa *oil may influence salivary gland function [11]. In a murine model of radiation-induced salivary gland injury, *N. sativa* oil administration helped preserve salivary output and promoted recovery of salivary gland stem cell populations compared with untreated controls [11]. While this study focused on long-term salivary gland recovery rather than the acute response observed in our investigation, it provides supporting evidence that *N. sativa *oil may have effects on pathways involved in salivary gland function [11]. Collectively, these findings and our observed early increase in salivary volume suggest that *N. sativa* oil may influence salivary physiology through both immediate secretory responses and potential longer-term effects on glandular health.

An additional finding of interest was the marked increase in average salivary protein concentration following *N. sativa *oil rinsing; however, this increase should be interpreted with caution due to the lack of inferential statistical analyses. Across all rinse durations, protein concentrations were approximately 2.5- to 5-fold higher than those observed following saline rinsing. The greatest increase was observed after the one-minute rinse duration, paralleling the period in which the largest increase in salivary volume was also detected.

Overall, this investigation demonstrates that rinse duration significantly influences salivary pH and volume, while *N. sativa* oil rinsing produces a distinct pattern of salivary volume changes compared with saline, particularly during early exposure. Furthermore, *N. sativa *oil rinsing was associated with higher salivary protein concentrations; however, these findings require cautious interpretation. These observations provide preliminary evidence that *N. sativa* oil may influence multiple components of salivary physiology involved in maintaining oral homeostasis.

Limitations and future directions

Several limitations should be considered when interpreting the findings of this pilot study. The study included a relatively small sample size (n = 10), which limits statistical power and reduces the ability to detect subtle treatment-related effects. The investigation employed a non-randomized, fixed design in which all participants completed the saline rinse protocol before the *N. sativa* oil protocol. Consequently, potential sequence bias and order effects cannot be excluded. Furthermore, the study evaluated only the immediate short-term effects of rinsing and therefore does not provide information regarding the durability or long-term clinical significance of the observed changes. Additionally, salivary protein concentration data were available only as aggregate measurements, precluding inferential statistical analysis and limiting interpretation of this outcome. The study population also consisted of generally healthy individuals, and the findings may not be directly generalizable to patients with salivary gland dysfunction, xerostomia, periodontal disease, or other oral pathologies.

Other limitations, including the small sample size and the absence of an a priori power calculation, may limit the generalizability of the findings. Additionally, the broad age range and unequal sex distribution reflected recruitment availability, and the study was not designed to assess age- or sex-related differences in salivary parameters. Future studies with larger, balanced cohorts are warranted. The lack of blinding represents a potential limitation; however, objective laboratory-based measurements were performed using standardized methods to reduce variability.

Future investigations should incorporate larger, randomized controlled study designs to confirm the findings observed in this pilot study and minimize potential sequence-related bias. Longitudinal studies are also warranted to determine whether the observed effects on salivary volume, pH, and protein concentration are sustained over time and translate into measurable improvements in oral health outcomes. Furthermore, comprehensive biochemical and proteomic analyses of saliva may help identify specific salivary proteins and molecular pathways influenced by *N. sativa*, thereby providing greater insight into its potential mechanisms of action and therapeutic relevance in oral medicine.

## Conclusions

The present pilot study demonstrated that rinse duration significantly affects salivary pH and volume, while *N. sativa* oil rinsing produced a distinct salivary volume response characterized by a significant early increase in secretion following the one-minute rinse. Additionally, *N. sativa* oil rinsing was associated with higher salivary protein concentrations, although these findings should be interpreted cautiously due to the lack of inferential statistical analysis. Overall, these findings provide preliminary evidence that *N. sativa* oil may influence multiple aspects of salivary physiology and support further investigation into its potential role in oral health.

## Appendices

Shapiro-Wilk normality test results for salivary pH and volume data

Appendix 1

**Table 9:** Shapiro–Wilk normality test results for salivary pH measurements across treatment conditions and rinse durations. Normality was assessed using the Shapiro–Wilk test with α = 0.05. A p-value >0.05 indicates that the data did not significantly deviate from a normal distribution.

Treatment condition	Rinse Duration	Shapiro–Wilk W	P-value	Normality assumption (α = 0.05)
Saline	1 minute	0.9491	0.6576	Passed
Saline	5 minutes	0.9293	0.4414	Passed
Saline	10 minutes	0.9293	0.4414	Passed
*Nigella sativa* oil	1 minute	0.7186	0.0015	Not passed
*Nigella sativa *oil	5 minutes	0.9519	0.6914	Passed
*Nigella sativa* oil	10 minutes	0.9447	0.6066	Passed

Appendix 2 

**Table 10:** Shapiro–Wilk normality assessment of log-transformed salivary volume across treatment conditions and rinse durations. Normality was assessed using the Shapiro–Wilk test following log transformation of salivary volume data. A p-value >0.05 indicates no significant deviation from a normal distribution.

Treatment condition	Rinse duration	Shapiro–Wilk W	P-value	Normality assumption (α = 0.05)
Saline	1 minute	0.9295	0.4433	Passed
Saline	5 minutes	0.9094	0.2771	Passed
Saline	10 minutes	0.8636	0.0840	Passed
*Nigella sativa *oil	1 minute	0.9218	0.3722	Passed
*Nigella sativa* oil	5 minutes	0.9299	0.4473	Passed
*Nigella sativa* oil	10 minutes	0.9342	0.4905	Passed

## References

[1] Dawes C, Pedersen AM, Villa A (2015). The functions of human saliva: a review sponsored by the World Workshop on Oral Medicine VI. Arch Oral Biol.

[2] Uchida H, Ovitt CE (2022). Novel impacts of saliva with regard to oral health. J Prosthet Dent.

[3] Okuyama K, Yanamoto S (2024). Saliva in balancing oral and systemic health, oral cancer, and beyond: a narrative review. Cancers (Basel).

[4] Matsuoka M, Soria SA, Pires JR, Sant'Ana AC, Freire M (2025). Natural and induced immune responses in oral cavity and saliva. BMC Immunol.

[5] Milleman K, Bosma ML, Saito A (2025). Comparison of safety, salivary flow rate, and pH with different mouthrinse formulations in addition to toothbrushing vs toothbrushing only: results from a 2-week phase 4 randomized controlled clinical trial. J Am Dent Assoc.

[6] Peedikayil FC, Diwakar N, Chandru TP, Kottayi S, Vardhanan YS, Narasimhan D (2022). Effect of oil pulling on pH, buffering capacity and total antioxidant capacity of saliva in children: a randomised control study. J Clin Diagn Res.

[7] Griessl T, Zechel-Gran S, Olejniczak S, Weigel M, Hain T, Domann E (2021). High-resolution taxonomic examination of the oral microbiome after oil pulling with standardized sunflower seed oil and healthy participants: a pilot study. Clin Oral Investig.

[8] Almaghlouth A, Shaheen A, Alkhamis H, Alswied R, Aloraini N (2023). The impact of Nigella sativa on oral health: a scoping review. J Med Law Public Health.

[9] Mekhemar M, Hassan Y, Dörfer C (2020). Nigella sativa and thymoquinone: a natural blessing for periodontal therapy. Antioxidants (Basel).

[10] Mustapha I, Mustamand M, Caesar D, Gu X (2026). Preliminary in vitro evaluation of the growth-inhibitory effects of Nigella sativa oil on Streptococcus mutans. Cureus.

[11] Luff M, Evans L, Hiyari S (2023). Nigella sativa oil mitigates xerostomia and preserves salivary function in radiotherapy-treated mice. Laryngoscope Investig Otolaryngol.

[12] Setiawatie EM, Gani MA, Rahayu RP, Ulfah N, Kurnia S, Augustina EF, Sari DS (2022). Nigella sativa toothpaste promotes anti-inflammatory and anti-destructive effects in a rat model of periodontitis. Arch Oral Biol.

[13] Alabdullah M, Kara Beit ZZ, Shehada A (2023). Comparative clinical study of the effect of Nigella sativa oil on soft tissue healing and inflammation reduction compared to eugenol in the context of dry socket. Cureus.

[14] Al-Attass SA, Zahran FM, Turkistany SA (2016). Nigella sativa and its active constituent thymoquinone in oral health. Saudi Med J.

[15] Pedersen AM, Sørensen CE, Proctor GB, Carpenter GH, Ekström J (2018). Salivary secretion in health and disease. J Oral Rehabil.

[16] Buzalaf MA, Hannas AR, Kato MT (2012). Saliva and dental erosion. J Appl Oral Sci.

[17] Arthy K, Sri Chinthu KK, Prasad H, Rajmohan M, Prema P, Shanmuganathan S (2026). Effect of cumin, black cumin and fennel seeds on pH of saliva - an experimental study. Oral Maxillofac Pathol J.

[18] Dagtas S, Griffin RJ (2021). Nigella sativa extract kills pre-malignant and malignant oral squamous cell carcinoma cells. J Herb Med.

[19] Proctor GB (2016). The physiology of salivary secretion. Periodontol 2000.

[20] Houghton JW, Carpenter G, Hans J, Pesaro M, Lynham S, Proctor G (2020). Agonists of orally expressed TRP channels stimulate salivary secretion and modify the salivary proteome. Mol Cell Proteomics.

